# Effect of Probiotic Lactobacillus salivarius on Peri-Implantitis Pathogenic Bacteria: An In Vitro Study

**DOI:** 10.7759/cureus.20808

**Published:** 2021-12-29

**Authors:** Munaz Mulla, Shashikanth Hegde, Ajit Koshy, Mushir Mulla

**Affiliations:** 1 Periodontology, Yenepoya University, Mangalore, IND; 2 Oral Pathology and Microbiology, M.A. Rangoonwala College of Dental Sciences and Research Centre, Pune, IND; 3 Oral and Dental Health, College of Applied Health Sciences, Qassim University, Arrass, SAU

**Keywords:** invitro, minimum inhibitory concentration, lactobacillus salivarius, probiotics, peri-implantitis

## Abstract

Background

Varied treatment modalities have been described in the past for the management of peri-implant diseases but the evidence of the use of probiotics for the treatment of peri-implantitis is limited. The aim of this study was to determine the antagonistic growth effects of *Lactobacillus salivarius* on the growth of peri-implantitis pathogens.

Material and method

Anin vitroassessment of probiotic *L. salivarius *on peri-implantitis pathogens was done using the serial tube dilution method. Minimum inhibitory concentration was calculated for five subgingival pathogens namely *Porphyromonas gingivalis*, *Aggregatibacter actinomycetemcomitans*, *Prevotella intermedia*, *Streptococcus salivarius*, and *Staphylococcus aureus. *Minimum inhibitory concentration (MIC) is defined as the lowermost concentration of any drug that prevents the noticeable growth of the test organism. In vitro assessment to determine the MIC is necessary for an in vivo application. The MIC value will also help to find the drug’s accurate dosage.

Results

Peri-implantitis pathogens were cultured from individuals diagnosed with peri-implantitis. Except for *A. actinomycetemcomitans,* all other pathogens were susceptible to the probiotic. *S. salivarius* had the lowest MIC (0.8mg/mL).

Conclusion

The MIC value for pathogens will help to determine the effective mode and form of probiotic that can be used for the treatment of peri-implantitis.

## Introduction

The term "Probiotic" is used to classify those substances that are released by one organism to enhance the growth of another [1], and is coined after the Greek word bio-tikos which means “for life”. They are considered beneficial for the health when taken in adequate amounts [2]. Since its initial use, several authors have found a positive correlation between probiotics and gut health, oral health, halitosis, dental caries, and oral candidiasis [3-5]. Periodontal inflammation has also benefited from the use of probiotics. Various *Lactobacilli* strains have been studied in the past. *Lactobacillus acidophilus* strain was found to be beneficial when used in patients having gingivitis, periodontitis, and pregnancy-induced gingivitis [6]. *Lactobacillus brevis* and *Lactobacillus reuteri* have also been shown to improve gingival bleeding in individuals [7,8]. *Lactobacillus salivarius* was found to reduce the gingival probing depth and also reduce the periodontal pathogens in dental plaque [9]. The successful results of probiotics in periodontal therapy have given rise to exploring their beneficial role in peri-implant diseases. Peri-implant mucositis and peri-implantitis constitute peri-implant diseases. Peri-implant mucositis is an inflammatory condition without bone loss and is limited to the surrounding tissues of the implant. On the contrary, peri-implantitis is associated with the loss of supporting bone [10]. Peri-implantitis microflora is more complex, consisting of mainly anaerobic gram-negative bacteria. When compared to periodontitis, tissue destruction was significantly higher among individuals with peri-implantitis. Although various treatment options are available, limited evidence is present on the usage of probiotics for the treatment of peri-implant diseases [11,12]. Thus, this study was carried out to evaluate the in vitro effect of probiotic *L. salivarius* on peri-implantitis pathogens. The aim of this study was to demonstrate the antagonistic growth effects of probiotic *L. salivarius* on growth suppression of peri-implantitis pathogens, such as *Porphyromonas gingivalis*, *Aggregatibacter actinomycetemcomitans*, *Prevotella intermedia*, *Streptococcus salivarius*, and *Staphylococcus aureus*, using the serial tube dilution method.

## Materials and methods

This study was carried out at M.A. Rangoonwala College of Dental Sciences and Research Centre, Pune. Ethical clearance was obtained from the institutional ethical committee (Reference number: MCES/EC/Perio.PhD/349-A/2016). Patients reporting to the department of periodontology and diagnosed with peri-implantitis were recruited for this study and selected if they complied with the selection criteria. The inclusion criteria included patients who were 18 years or older with a minimum of one implant having peri-implantitis. Peri-implantitis was diagnosed if the probing depth around the implant was ≥ 4 mm, bleeding on probing, loss of supporting bone when viewed on radiographs, and with no implant mobility. Patients consuming tobacco, with systemic diseases, and/or under medication for systemic health were excluded. All subjects provided appropriate informed consent according to the guidelines of the Helsinki Declaration.

After the patients were selected for the study, scaling was done to remove supra-gingival plaque. Subgingival plaque samples were then collected to obtain the peri-implantitis pathogens. Sterile paper point (30no.) was used for the same. After keeping it in the peri-implant pocket for 30 seconds, it was transferred instantly into a sterile Eppendorf Tube® (Eppendorf Corp., Hamburg, Germany). A transport medium containing thioglycollate broth, 2 ml (0.4% agar, 0.15% thioglycollate buffered saline) was used for the same. Once the sample was collected, it was sent to the microbiological lab for further investigation. Each collected sample was incubated under aerobic and anaerobic conditions to identify five peri-implant pathogens: *P. gingivalis, A. actinomycetemcomitans, P. intermedia, S. salivaruis*, and *S. aureus*. To support the growth of the bacteria, various culture media were used. Aerobes: Blood Agar and MacConkey Agar, Anaerobes: Blood Agar, and Bacteroides Bile Esculin (BBE) Agar. 

*L. Salivarius* was procured from Agharkar Research Institute, Pune, and was cultured according to their recommendation. Rogosa agar (selective medium) was used to culture *L. Salivarius*, with an incubation period of three to four days at 37°C. Bergey's Manual® of Systematic Bacteriology (Springer: New York) was taken as a reference for identification of the colonies based on their colony, biochemical, and morphological characteristics. Minimum inhibitory concentration (MIC) by means of the serial tube dilution method was employed to measure the effect of probiotic *L. Salivarius* [13]. For initial preparation, 20μl of lactobacilli strain was mixed with the 380μl of Thioglycollate broth to make a volume of 400μl. The first dilution was prepared by adding 200μl from this tube into a separate test tube containing 200μl of Thioglycollate broth. This was termed as 10−1 dilution. To make the next dilution, 200μl was added from this 10-1 to a test tube containing 200μl of Thioglycollate broth. This was termed as 10-2 dilution. Similarly, a total of nine dilutions were prepared. Culture suspensions of the five peri-implantitis pathogens were made by adding 5μl from their maintained stock cultures into 2ml of Thioglycollate broth. From this, 200μl was added into each serially diluted tube. All these tubes were then incubated in an anaerobic jar for 48-72 hours at 37°C. The presence of any turbidity indicated the growth of the organism. The tube that contained the least concentration of the lactobacilli strain with no turbidity was regarded as the MIC for that particular microorganism.

## Results

Subgingival plaque samples were collected to obtain five different pathogens namely: *S. aureus, S. salivaris, P. intermedia, A. actinomycetemcomitans*, and *P. gingivalis*. These pathogens were tested against probiotic *L. Salivarius* to determine the MIC values (Table 1).

**Table 1:** The effect of Lactobacillus salivarius concentrations on various peri-implantitis pathogens S: Sensitive; R: Resistant; PG: *Porphyromonas gingivalis*; AA*: Aggregatibacter actinomycetemcomitans*; PI*: Prevotella intermedia*; S.SAL*: Streptococcus salivaris*; STAPH.A*: Staphylococcus aureus*

Probiotic concentrations		100 mg/ml	50 mg/ml	25 mg/ml	12.5 mg/ml	6.25 mg/ml	3.12 mg/ml	1.6 mg/ml	0.8 mg/ml	0.4 mg/ml	0.2 mg/ml
PG		S	S	R	R	R	R	R	R	R	R
PI		S	S	R	R	R	R	R	R	R	R
S.SAL		S	S	S	S	S	S	S	S	R	R
STAPH.A		S	S	S	R	R	R	R	R	R	R
AA		R	R	R	R	R	R	R	R	R	R

Nine concentrations of the probiotics were used to calculate the MIC. In the present study, *P. gingivalis, P. intermedia*, *S. salivaris*, and *S. aureus* were sensitive to *L. salivarius*. *P. gingivalis* was sensitive until 50mg/mL and showed resistance to further dilution thereby indicating its MIC. Similarly, MIC for *P. intermedia* was 50mg/mL, for *S. salivaris* was 0.8mg/mL, and *S. aureus* was 25mg/ml. However, for *A. actinomycetemcomitans*, the performed dilutions did not show sensitivity.

## Discussion

In peri-implantitis, there is progressive destruction of the hard and soft tissues surrounding the implant [14]. Peri-implantitis is known as a multi-factorial disease with numerous risk factors for the same. Various pathogens are also associated with the progression of peri-implantitis. In the study by Persson et al., the pathogens found to be associated with peri-implantitis were *Treponema denticola, Tannerella forsythia, Streptococcus mitis, Streptococcus intermedius, S. aureus, P. gingivalis, Haemophilus influenzae, Helicobacter pylori, Campylobacter rectus, A. actinomycetemcomitans* [15].

Progression of peri-implantitis leads to excessive loss of supporting tissues causing implant failure or loss of the implant. The treatment modalities of peri-implantitis vary from non-surgical therapy, surgical therapy, and local and systemic antimicrobial therapy. Yet, some patients do not show any response to any of the treatments [16]. Also, antibiotic resistance limits the use of antibiotics in these cases [17,18]. Therefore, probiotics as an adjunct therapy in the management of peri-implantitis is studied in recent years. The use of probiotics in treating caries, halitosis, gingival and periodontal diseases have been reported in the past, but very little literature is available on the use of probiotics in treating peri-implantitis. 

For this study, five pathogens were selected that were known to be present predominantly in peri-implantitis sites (*P. gingivalis, A. actinomycetemcomitans, P. intermedia, S. salivarius*, and *S. aureus*). These pathogens were obtained from the patients who were diagnosed with peri-implantitis. They were then tested against the antagonistic effect of *L. salivarius*.

*L. salivarius* is amongst the major species in human saliva [19,20]. Their property includes the production of organic acids from the fermentation of carbohydrates, thereby interfering with the growth of other neighboring microorganisms [21]. Because of this antagonistic property they can be used to combat the spread of infection and improve the host immunity. Authors have suggested their beneficial role when used to treat periodontal and peri-implant diseases [19]. Thus, for this study, *L. salivarius* was used to test its suppressive effect against the peri-implantitis pathogens. 

Based on the susceptibility of the microorganisms, the MIC can be low or high. The MIC is defined as the lowermost concentration of a drug that, after overnight incubation, will suppress the growth of an organism [13]. In this present study, *S. salivarius* had the lowest MIC (0.8mg/mL) whereas *P. gingivalis* and *P. intermedia* had higher MIC (50mg/mL). Similarly, previous authors have also reported in vitro effect of *L. salivarius* on *P. gingivalis*, *P. intermedia*, and *Prevotella nigrescens* [22]. *L. salivarius* has also been shown to decrease pocket probing depth and plaque index in people diagnosed with periodontal disease [23,24]. *L. salivarius* and *Lactobacillus fermentum* and their concentrated fermentative broth inhibited the growth of *P. gingivalis, Streptococcus sanguis*, and *Streptococcus mutans* [25]. *A. actinomycetemcomitans* was not found to be susceptible to *L. salivarius* in this present study. Thus, to identify the precise value of MIC for *A. actinomycetemcomitans*, additional dilutions of >100 mg/mL are required.

## Conclusions

Currently. there is insufficient data to demonstrate the effective usage of probiotics in the management of peri-implantitis. This study identifies the susceptibility of various peri-implantitis pathogens to *L. salivarius* by providing their MIC values. According to the authors based on the results obtained, a concentration of 50mg/ml of probiotic* L.salivarius *can be effectively used against *P.gingivalis, P.intermedia*, *S.salivaris, *and *S.aureus* in the management of periimplantitis. This can help us to detect the ideal dosage and formulation required for antagonistic activity of *L. salivarius* to treat peri-implantitis. Further research needs to be conducted to identify the effective form of probiotics that can be used and also the effective way to administer these probiotics to obtain the maximum benefit in treating peri-implantitis.

## References

[1] Lilly DM, Stillwell RH (1965). Probiotics: growth-promoting factors produced by microorganisms. Science.

[2] (2002). Guidelines for the evaluation of probiotics in food: report of a joint FAO/WHO working group on drafting guidelines for the evaluation of probiotics in food. Guidelines for the evaluation of probiotics in food: report of a joint FAO/WHO working group on drafting guidelines for the evaluation of probiotics in food.

[3] Comelli EM, Guggenheim B, Stingele F, Neeser JR (2002). Selection of dairy bacterial strains as probiotics for oral health. Eur J Oral Sci.

[4] Iwamoto T, Suzuki N, Tanabe K, Takeshita T, Hirofuji T (2010). Effects of probiotic Lactobacillus salivarius WB21 on halitosis and oral health: an open-label pilot trial. Oral Surg Oral Med Oral Pathol Oral Radiol Endod.

[5] Gruner D, Paris S, Schwendicke F (2016). Probiotics for managing caries and periodontitis: systematic review and meta-analysis. J Dent.

[6] Zhao JJ, Feng XP, Zhang XL, Le KY (2012). Effect of Porphyromonas gingivalis and Lactobacillus acidophilus on secretion of IL1B, IL6, and IL8 by gingival epithelial cells. Inflammation.

[7] Schlagenhauf U, Jakob L, Eigenthaler M, Segerer S, Jockel-Schneider Y, Rehn M (2016). Regular consumption of Lactobacillus reuteri-containing lozenges reduces pregnancy gingivitis: an RCT. J Clin Periodontol.

[8] Maekawa T, Hajishengallis G (2014). Topical treatment with probiotic Lactobacillus brevis CD2 inhibits experimental periodontal inflammation and bone loss. J Periodontal Res.

[9] Nishihara T, Suzuki N, Yoneda M, Hirofuji T (2014). Effects of Lactobacillus salivarius-containing tablets on caries risk factors: a randomized open-label clinical trial. BMC Oral Health.

[10] Poli PP, Cicciu M, Beretta M, Maiorana C (2017). Peri-implant mucositis and peri-implantitis: a current understanding of their diagnosis, clinical implications, and a report of treatment using a combined therapy approach. J Oral Implantol.

[11] Mulla M, Mulla M, Hegde S, Koshy AV (2020). Management of peri-implantitis—a narrative review. J Crit Rev.

[12] Mulla M, Mulla M, Hegde S, Koshy AV (2021). In vitro assessment of the effect of probiotic lactobacillus reuteri on peri-implantitis microflora. BMC Oral Health.

[13] Andrews JM (2001). Determination of minimum inhibitory concentrations. J Antimicrob Chemother.

[14] Farhan LS (2018). The microbial etiology and pathogenesis of peri-implantitis. Oral Health Dent Manag.

[15] Persson GR, Renvert S (2014). Cluster of bacteria associated with peri-implantitis. Clin Implant Dent Relat Res.

[16] Wasserman B, Hirschfeld L (1988). The relationship of initial clinical parameters to the long-term response in 112 cases of periodontal disease. J Clin Periodontol.

[17] van Winkelhoff AJ, Herrera D, Winkel EG, Dellemijn-Kippuw N, Vandenbroucke-Grauls CM, Sanz M (1999). Antibiotic resistance in the subgingival microflora in patients with adult periodontitis. A comparative survey between Spain and the Netherlands [Article in Dutch]. Ned Tijdschr Tandheelkd.

[18] Mulla M, Mulla M, Kashyap R, Hegde S, Maiya A, Sarpangala M, Sayed FR (2020). Evaluation of the efficacy of a dentifrice containing amine fluoride on gingival status—a clinical investigation. Int J Pharm Res.

[19] Hojo K, Mizoguchi C, Taketomo N, Ohshima T, Gomi K, Arai T, Maeda N (2007). Distribution of salivary Lactobacillus and Bifidobacterium species in periodontal health and disease. Biosci Biotechnol Biochem.

[20] Kõll-Klais P, Mändar R, Leibur E, Marcotte H, Hammarström L, Mikelsaar M (2005). Oral lactobacilli in chronic periodontitis and periodontal health: species composition and antimicrobial activity. Oral Microbiol Immunol.

[21] McGroarty JA, Tomeczek L, Pond DG, Reid G, Bruce AW (1992). Hydrogen peroxide production by Lactobacillus species: correlation with susceptibility to the spermicidal compound nonoxynol-9. J Infect Dis.

[22] Ishikawa H, Aiba Y, Nakanishi M, Oh-hashi Y, Koga Y (2003). Suppression of periodontal pathogenic bacteria by the administration of Lactobacillus salivarius T12711. J Jap Soc Periodontol.

[23] Shimauchi H, Mayanagi G, Nakaya S, Minamibuchi M, Ito Y, Yamaki K, Hirata H (2008). Improvement of periodontal condition by probiotics with Lactobacillus salivarius WB21: a randomized, double-blind, placebo-controlled study. J Clin Periodontol.

[24] Gao J, Yu S, Zhu X, Yan Y, Zhang Y, Pei D (2020). Does probiotic Lactobacillus have an adjunctive effect in the nonsurgical treatment of peri-implant diseases? A systematic review and meta-analysis. J Evid Based Dent Pract.

[25] Chen LJ, Tsai HT, Chen WJ (2012). In vitro antagonistic growth effects of Lactobacillus fermentum and lactobacillus salivarius and their fermentative broth on periodontal pathogens. Braz J Microbiol.

